# Visualizing a Single‐Crystal‐to‐Single‐Crystal [2+2] Photodimerization through its Lattice Dynamics: An Experimental and Theoretical Investigation

**DOI:** 10.1002/cphc.202200168

**Published:** 2022-04-29

**Authors:** Andrea Giunchi, Lorenzo Pandolfi, Tommaso Salzillo, Aldo Brillante, Raffaele G. Della Valle, Simone d'Agostino, Elisabetta Venuti

**Affiliations:** ^1^ Dipartimento di Chimica Industriale “Toso Montanari” Università di Bologna viale del Risorgimento, 4 40136 Bologna Italy; ^2^ Dipartimento di Chimica “Giacomo Ciamician” Università di Bologna via Francesco Selmi, 2 40126 Bologna Italy

**Keywords:** photodimerization, 4-aminocinnamic acid, Raman spectroscopy, lattice dynamics, density functional theory

## Abstract

In homogeneous solid‐state reactions, the single‐crystal nature of the starting material remains unchanged, and the system evolves seamlessly through a series of solid solutions of reactant and product. Among [2+2] photodimerizations of cinnamic acid derivatives in the solid state, those involving salts of the 4‐aminocinnamic acid have been recognized to proceed homogeneously in a “single‐crystal‐to‐single‐crystal” fashion by X‐ray diffraction techniques. Here the bromide salt of this compound class is taken as a model system in a Raman spectroscopy study at low wavelengths, to understand how such a mechanism defines the trend of the crystal lattice vibrations during the reaction. Vibrational mode calculations, based on dispersion corrected DFT simulations of the crystal lattices involved in the transformation, have assisted the interpretation of the experiments. Such an approach has allowed us to clarify the spectral signatures and to establish a correlation between the dynamics of the monomer and dimer systems in a process where chemical progress and crystal structural changes are demonstrated to occur simultaneously.

## Graphical abstract

**The single‐crystal‐to‐single‐crystal photodimerization** of the bromide salt of the 4‐aminocinnamic acid, taken as a model system for this class of reactions, is demonstrated to display a clear spectral signature in a low‐wavenumber Raman investigation assisted by dispersion‐corrected DFT simulations.

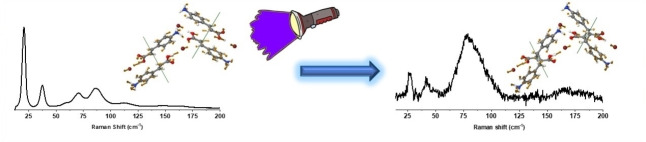

## Introduction

In single‐crystal‐to‐single‐crystal (SCSC) photoinduced reactions, crystalline morphology and structural order are maintained throughout the course of the transformation from reactant to product. Promising candidates for this kind of reaction are the [2+2] dimerizations of cinnamic acid derivatives, where the original formulation of Schmidt and Cohen's topochemical principle is satisfied[^1^, ^2^, ^3^] and the symmetry of the monomer packing correlates to that of the dimer and to the dimer's molecular symmetry. Despite meeting these requirements, the crystals often crack and shatter under irradiation. This phenomenon can possibly be prevented by adopting the strategy of the end‐tail irradiation suggested by Enkelmann,^[4]^ which leads to a more homogeneous distribution of the excited species within the crystal bulk. Consequently, even photochemical reactions that in most conditions would not occur as SCSC can be led to behave as such. Furthermore, the irradiation of micro‐crystals, rather than macro samples, avoids the co‐existence of transformed and untransformed crystal portions and the resulting build‐up of internal mechanical stress, which leads to sample disruption.

SCSC transformations represent a wonderful mean to understand the whole evolution of the reaction, as they link its progress to the crystal structural changes, and, quoting Naumov and Bharadwaj^[5]^ are “the dream of every crystallographer”. From an experimental point of view, they can be detected by the application of dynamic X‐ray crystal structure determination, by recording the smooth transition of the reactant diffraction pattern to that of the product and refining the occupational parameters to determine the yield so to obtain different structures^[6]^ and get the picture of the average situation of what is happening in the crystal as the reaction proceeds.

The study of the entire span of a SCSC process constitutes also an interesting and challenging opportunity for a spectroscopic study which aims at analyzing, in addition to the progress of the chemical event, also the vibrational modes which are representative of the evolution dynamics of the lattice. In a molecular crystal these are the collective motions which arise from the crystalline periodical arrangement of the molecules and to large extent depend on the intermolecular force field. Known as lattice phonons, they occur in the low energy region of the vibrational crystal spectrum and can in fact be used as lattice dynamics markers or structure fingerprints.

All solid‐state photochemical reactions begin by the formation of a solid solution of the product in the lattice of the reactant.[^1^, ^7^, ^8^] The possible fate of the initial solid solution on reaction progress has been the object of the seminal work of photocrystallography[^1^, ^7^] and has been summarized in the work by de Loera *et al*.,^[8]^ which indicates as possible outcomes the persistence of the same phase, the formation of a new stable or metastable phase, melting or amorphization. In the so‐called SCSC homogeneous systems, reactant and product persist in the same phase. Such a mechanism is made possible by the preconditions of isomorphism of the lattices of the two species and the similarities of their molecular structures.

When a reaction such as a dimerization proceeds in a SCSC fashion and the state of solid solution is maintained throughout the entire process, at any time the dimer is statistically distributed inside a lattice which is gradually transforming into the final one. Note that the prerequisite of molecular structure similarity is satisfied as each dimeric unit correlates to two monomers, and this also leads to crystal isomorphism. This kind of mechanism has been detected and widely described by X‐ray techniques (see references [6, 9] for an exhaustive number of cases), but much less attention has been dedicated to the description of the dynamic evolution of the lattice itself by spectroscopic methods, with the exception of some early works.[^10^, ^11^]

Lattice phonon measurements by low frequency Raman microscopy have been employed to probe the co‐existence of the two phases in heterogeneous photodimerization processes[^12^, ^13^, ^14^, ^15^, ^16^] in which the product separates from the reactant and a phase boundary between the two is formed.[^1^, ^8^] The homogeneous case, however, is expected to hold different characteristics. The binary solid solution constitutes a single phase, in which upon irradiation a set of van der Waals bonds get progressively and continuously replaced by covalent bonds. Because of this, vibrational modes that in the reactant can be described as motions of the monomers (lattice vibrations) in the product become relative motions of the two monomeric halves of the dimer (intra‐molecular vibrations). At any time during the reaction, which may or may not proceed up to a 100 % yield, very similar atomic motions would thus correspond to either lattice or intra‐molecular vibrations in different lattice points. This change in the nature of the vibrations would be completely unambiguous if one could assume a complete decoupling between molecular deformations (intra‐molecular vibrations) and displacements of the molecular skeletons as a whole (lattice vibrations). If all intra‐molecular degrees of freedom could also be ignored altogether, this would correspond to the so‐called rigid molecule approximation (RMA), often applied to the interpretation of the vibrational spectra of molecular crystals but clearly inapplicable in this case. Instead, it seems appropriate to use a reference description of the “RMA separation”, in which the intra and intermolecular degrees of freedom are all included and allowed to interact but are analyzed separately. We will see that this approach is indeed appropriate and useful.

In an alternative description that focuses on the crystal structure, the formation of the product molecules in the monomer lattice while the system remains in a single phase could be interpreted as the occurrence of static disorder that breaks down the periodicity of the system and disrupts the correlation lengths ruling the crystal dynamics. This, for instance, would account for the frequent band broadening, as disorder on such short length scales is likely to cause a partial non‐compliance of the **k**≃0 selection rule of the pure lattice.[^17^, ^18^]

Recently, a few works have reported the activation of the solid‐state photoreactivity in 4‐aminocinnamic acid (**1**) through formation of molecular salts of general formula [**1**H]A, that can be synthesized by reacting the parent compound with a series of inorganic acids HA.[^19^, ^20^, ^21^, ^22^] In particular, it was found that in both the salts [**1**H]Cl and [**1**H]Br, obtained by reaction of **1** with hydrochloric and hydrobromic acids, respectively, [2+2] photoreactions are quantitative and occur in SCSC fashion.[^21^, ^23^]

In the work of Ref.^[22]^ we investigated the effect of composition on the kinetics of the photoreaction in the series [**1**H]Br_x_Cl_1−x_ (0<x<1) making use of Raman and IR spectra in the intramolecular energy range and unraveled the chemical information conveyed by the latter spectroscopic technique by adopting a principal component analysis (PCA) approach. We thus observed that the time‐evolution of the low‐wavenumber Raman spectrum was markedly different from that recorded for crystal‐to‐crystal heterogeneous reactions.[^12^, ^13^, ^14^, ^16^] The observed behaviour had been predicted in the past but, to the best of our knowledge, experimentally described only for one system.^[24]^

Intrigued by this evidence, in the present work we attempt a description of the phenomenon for the [**1**H]Br model system with the support of the calculation of the vibrational properties by dispersion‐corrected DFT simulations of both reactant and product crystals. The aim is to get an understanding of the meaning of the lattice phonons in a molecular crystal made of a solid solution. Noticeably, DFT simulations of perfect crystals are performed using periodic boundary conditions, which imply considering only the atoms contained in the crystal unit cell. The lack of periodicity characterizing solid solutions makes the treatment of these phases more cumbersome because of the statistic distribution of the two components (monomer and dimer). Among the possible ways to tackle this problem, we have adopted the strategy of calculating phonon frequencies and intensities of the monomer in the lattice of the dimer and vice versa. This has allowed to track down how monomer lattice modes transform into the dimers’.

## Results and Discussion

[**1**H]Br crystal structure B is monoclinic P2_1_/c with Z=4 and cell parameters *a*=5.8723(3) Å, *b*=8.8268(6) Å, *c*=18.8691(15) Å, β=92.660(5)° and V=977.000(11) Å^3^, while [**1**_2_H_2_]Br_2_ is also monoclinic P2_1_/c con Z=2 and cell parameters *a*=5.9966(3) Å, *b*=8.7103(5) Å, *c*=17.850(2) Å, β=92.284(6)° and V=931.604 Å^3^. The structural relationship between monomer and dimer lattices and the similarities between their interactions patterns have been described in detail in Ref. [19]. In the monomer structure the pair of reacting molecules lie about a crystal inversion centre; accordingly, the dimer molecule is centrosymmetric.

The vibrational symmetry selection rules of the centrosymmetric space group P2_1_/c which determine the spectroscopic properties are the same for both structures: only the modes belonging to gerade (g) A_g_ and B_g_ symmetry representations can be observed by Raman spectroscopy.

Despite being so similar, the structures display in the lattice phonon region quite distinct vibrational spectral features, with an overall shift towards longer wavenumbers of the product spectrum resulting from the 4.6 % volume contraction of the unit cell upon dimerization.

**Figure 1:**
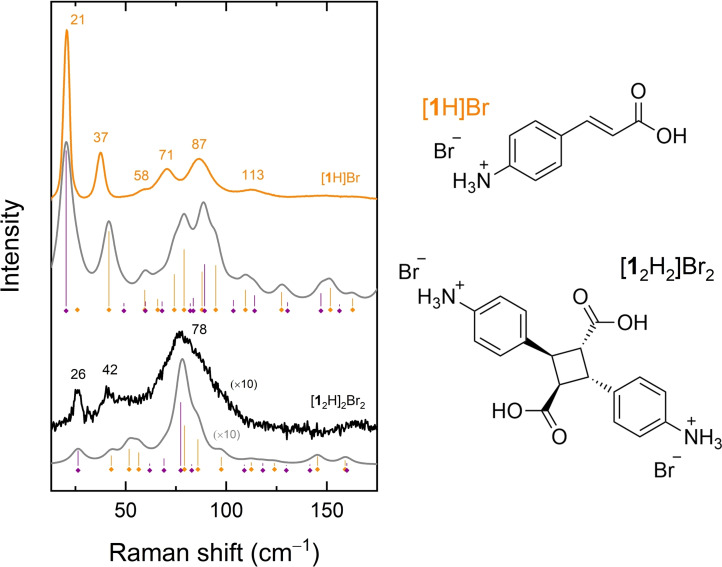
Reference Raman spectra in the energy range of the lattice phonon modes for the [**1**H]Br monomer (orange trace) and its [**1**_2_H_2_]Br_2_ photodimer (**black** trace) obtained by irradiation of a single crystal at λ=365 nm over a time span of ca 1 hour. The grey traces are the DFT simulated spectra for monomer (top) and dimer (bottom), respectively, calculated by assuming a lorentzian band shape with FWHM=4.5 cm^−1^. The vertical bars indicate the calculated positions of the peak values, with different colour codes to label the symmetry (yellow for A_g_ – violet for B_g_) of the mode. The molecular structures of the two compounds are also reported.

These characteristics are shown in Figure 1, where the Raman spectra of a [**1**H]Br single crystal before and after an irradiation time of ca 1 hour, sufficient to ensure a maximum conversion yield to the [**1**_2_H_2_]Br_2_ dimer, are reported in the low wavenumber range up to 175 cm^−1^, along with the corresponding DFT simulated spectra. The dimer spectrum coincides with that recorded after re‐crystallization and X‐ray analysis of a [**1**H]Br irradiated powder sample (Figure S1).

The agreement between experiments and calculations is good. For instance, the details of the first bands, i. e. those corresponding to a single mode rather than originating from the deconvolution of many scattering signals, are correctly reproduced, as is the observed strong intensity decrease of the entire spectrum of the dimer compared to that of the [**1**H]Br monomer.

In particular, the following main peaks can be identified and used as markers of the monomer spectrum: 21, 37, 58, 71, 87, 113 cm^−1^, as obtained by deconvolution. In the dimer spectrum, along with a narrower band at 26 cm^−1^, we observe a peak at 42 cm^−1^ and a broad band with maximum at 78 cm^−1^, while weaker spectral features appear at higher wavenumbers.

In analysing the vibrational properties of the two structures in the framework of RMA separation, we must consider that the unit cells of [**1**H]Br and [**1**_2_H_2_]Br_2_ contain the same number of atoms, so that the total number of degrees of freedom is the same, but with different distributions between inter‐ and intra‐molecular vibrations, arising from the presence of two distinct molecular species. This is bound to modify the coupling patterns, and accounts for frequency and intensity changes when the spectra of the two lattices are compared. It is necessary to check whether, despite its limits, a description based on RMA separation can be of support in establishing a correlation between the monomer and dimer crystal's dynamics.

If perfect RMA decoupling is applied, 18 and 12 Raman active pure lattice phonon modes would be expected for [**1**H]Br and [**1**_2_H_2_]Br_2_ crystals, which have 4 and 2 molecules per unit cell, respectively, and 4 anions. Many torsional modes as well as the internal rotations of the methyl groups fall in the low frequency spectrum of the cinnamic acid units of both compounds, hence in the crystal an effective mixing between these degrees of freedom and the lattice phonons might be foreseen. The contribution of the lattice phonons to a computed mode can be determined in the analysis of the DFT vibrational results via the (squared) projections of the mode eigenvector around and along the three molecular axes of inertia. Despite the coupling, out of the 26 modes calculated for the monomer crystal [**1**H]Br in the spectral window of Figure 1, below 130 cm^−1^ it is possible to identify many which can be described as having predominant (≥60 %) lattice phonon character, with those below 60 cm^−1^ being virtually pure combinations of rotations and translations of rigid molecules (Table S1). Note that the contribution to the mode translational components of the Br anions, calculated to vanish only above 130 cm^−1^, needs to be neglected in this analysis, centred on the vibrations of the bodies involved in the dimerization. It is thus possible to estimate the intermolecular character of each mode by focusing only on the motion of the molecular skeletons.

For the more flexible [**1**_2_H_2_]Br_2_ dimers, a neat separation between lattice and intramolecular modes is even more difficult. In the Raman active lattice modes, the motion of the centrosymmetric molecular skeletons possesses rotational character only, with the anions contributing to the translational part. Considering this, out of the 20 modes calculated over Figure 1 energy interval, at least 6 modes can be described as predominantly due to rigid motions of the molecular skeletons. Thus, also in the case of the flexible [**1**_2_H_2_]Br_2_, RMA separation still assists the spectra interpretation, even though the mixing with the intramolecular modes is more effective.

**Figure 2:**
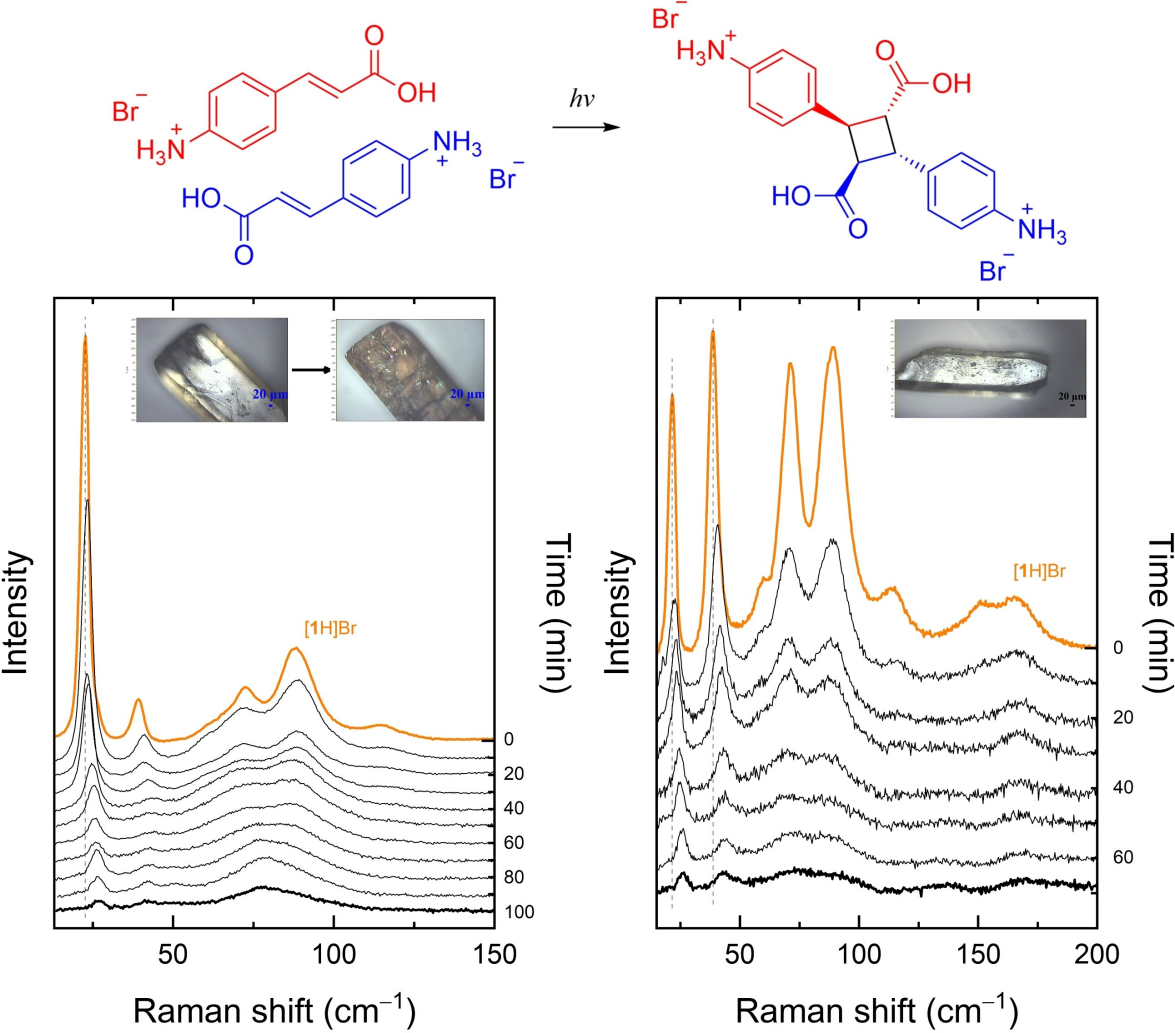
SCSC transformation of a single crystal of [**1**H]Br under irradiation (λ=365 nm) followed by Raman spectroscopy in the lattice phonon interval. Spectra were recorded at time intervals of 10′. The orange trace identifies the pure monomer [**1**H]Br; the **black** trace the pure dimer [**1**_2_H_2_]Br_2_. The vertical line highlights the red shift of the monomer lattice phonon band of lowest energy with the progression of the reaction.

Figure 2 displays the Raman spectra of the SCSC transformation followed stepwise in the low wavenumber range as a function of the irradiation time for two differently oriented [**1**H]Br single crystals, chosen in such a way to optimize the detection of all the observable bands, the intensity of which depends on their polarization properties. In agreement with the measurements on the recrystallized [**1**_2_H_2_]Br_2_ product,^[22]^ the overall intensity of the dimer's Raman band gets weaker with the progression of the reaction, and in addition the appearance of the fluorescence emission over the wavenumber interval of interest increases the spectra background. The intensity lowering of the dimer spectrum is recorded also by the DFT simulations, and therefore can be assumed to be an intrinsic effect, even though the simultaneous band broadening and the detected sample damaged surface suggest that some structural disorder occurs at the end of the reaction. In Figure S2 the corresponding lattice phonon Raman spectrum for the photodimerization reaction of the system [**1**H]Cl is also reported. Even though the transformation of [**1**H]Cl to its dimer takes place on longer time scales,^[22]^ the spectral trend displayed is the same as for [**1**H]Br.

The most relevant piece of information we gather from the data of Figure 2, however, is that the observed lattice phonon time evolution is unlike that of the high frequency region subjected to kinetic investigation in Ref. [22], which can be decomposed at any time as the overlap of the reactant and product spectral signals. Here the progress of the reaction is accounted for by the continuous evolution of the lattice phonon spectrum, which probes the change of composition of a single solid phase. Both structurally and dynamically, such a phase can be represented as composed of two almost identical components, i. e. the [**1**H]Br monomer itself and the monomeric half of the [**1**_2_H_2_]Br_2_ dimer. The Raman scattering, which initially contains the pure [**1**H]Br crystal, under irradiation weakens and monotonically transforms into the phonon spectrum of [**1**_2_H_2_]Br_2_, the molecules of which are gradually forming. The process is a kind of slow melting of one structure into the other.^[24]^ Such a spectral behavior is very different from what is observed in a heterogenous mechanism, in which the photoproduct forms as a guest in the host lattice of the reactant, but eventually crystallizes in its own structure in a phase separated from the parent one. In such a case the Raman spectrum records the existence of randomly distributed domains of two (or more) distinct chemical species and phases, so that also the low wavenumber region can be decomposed as the sum of the spectra of the lattices of the pure compounds.[^11^, ^12^, ^13^, ^14^, ^15^, ^16^]

By projecting the DFT eigenvectors of the [**1**H]Br crystal onto those of [**1**_2_H_2_]Br_2_ we can gather information on how the vibrational modes of one crystal map onto those of the other. For most modes we find that a one‐to‐one correspondence applies. A single mode in the [**1**H]Br crystal is thus representable as a single mode in the [**1**_2_H_2_]Br_2_ crystal, i. e. their overlap (or dot product) is very close to 1, and vice versa. As expected, the largest overlap involves modes with similar frequencies. Such a result is graphically reported in Figure S3, where the modes are ordered by their frequency and it is found that most of the overlap is on the diagonal, confirming a biunivocal correspondence.

More information can be obtained by analyzing in detail the eigenvector dot product of Table S2, where all the best correspondences between the [**1**H]Br and [**1**_2_H_2_]Br_2_ Raman modes are reported.

**Figure 3:**
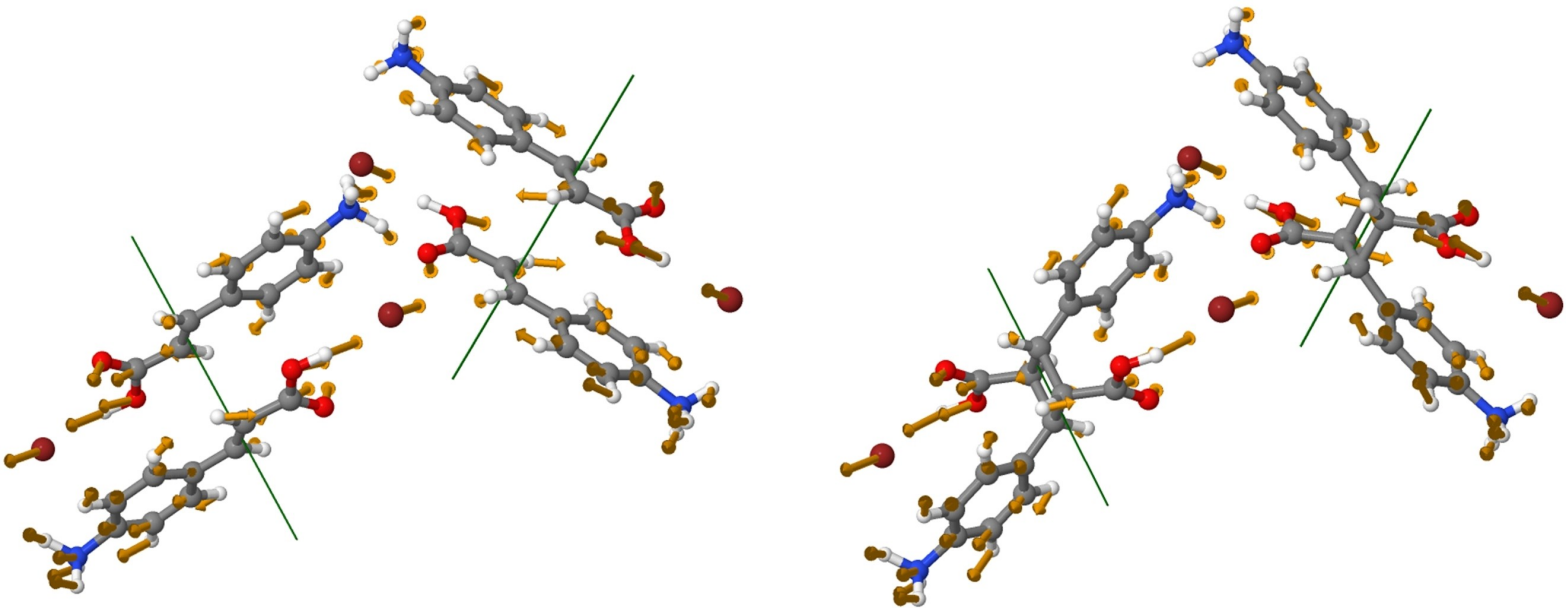
Graphic representation of the eigenvectors of the lowest energy lattice phonon mode for the monomer [**1**H]Br (left) and the dimer [**1**_2_H_2_]Br_2_ (right).

As an example, we look at the behavior of the B_g_ Raman active mode of the [**1**H]Br monomer lying at 21 cm^−1^ and calculated at 20.4 cm^−1^, which is found to fully retain its lattice phonon character in the course of the cell transformation to finally map onto the dimer 26 cm^−1^ mode. As shown by the graphic representation of Figure 3 (and the animation of the SI) the [**1**H]Br mode is described by the DFT simulations as the concerted rotation about an axis nearly parallel to the N inertia axis of each of the two pairs of monomer reactants pre‐arranged in the unit cell to give the dimers. Demonstrating that the interaction patterns within the reactant are appreciably maintained, the monomeric bound units display the same kind of displacement in the product lattice, where each pair has transformed into a dimer. This is a vibration which best corresponds to the definition of “spectra amalgamation” given in Ref. [10], where it is stated that whenever the difference between the host (monomer) and guest (dimer) pure crystal phonon frequencies is smaller than the phonon bandwidth, amalgamation of their phonons occurs.

Even though we cannot assume a kinetics analysis based on the behavior of a single band to be fully reliable, we observe that the amplitude and frequency of this lattice phonon can be mapped throughout the photoconversion and this suggests taking them as quantitative markers of the lattice transformation.

**Figure 4:**
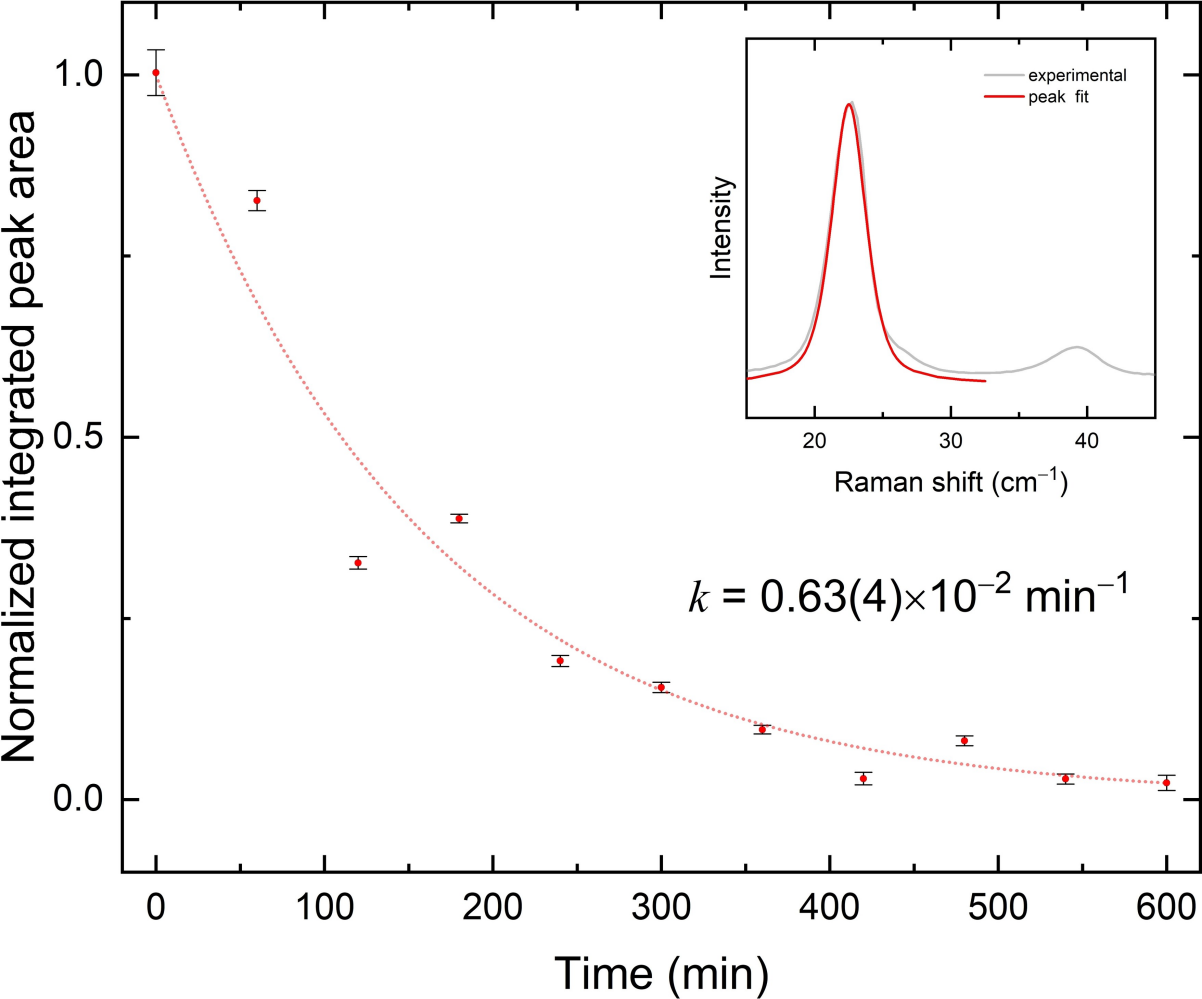
Amplitudes of the Raman lattice phonon band detected at 21 cm^−1^ in [**1**H]Br monomer reported as a function of the irradiation time (λ=365 nm). Integrated areas and corresponding errors were obtained via deconvolution with pseudo‐voigt band shapes.

Indeed, the fit of the integrated intensity of such a band *vs* irradiation time, as shown in Figure 4, yields an exponential decay, with a rate constant k=0.63(4)×10^−2^ min^−1^ for the [**1**H]Br salt. Notably, one cannot help noticing that the value of the constant is very close to what found from the ATR‐FTIR kinetic analysis by PCA on the single crystal of the same compound.^[22]^ This validates the assumption that the chemical reaction, monitored by the evolution of the intramolecular IR spectrum, and the lattice transformation, followed via the evolution of the intermolecular Raman spectrum, do occur simultaneously. It also represents a striking difference compared to crystal‐to‐crystal heterogenous reactions, for which Raman measurements spanning both spectral ranges[^13^, ^16^] have clearly demonstrated that the phase reconstruction takes place with a measurable time delay with respect to the chemical event.

Proceeding in the vibrational analysis, one finds that the computed A_g_ lattice modes at 26.3 and 41.7 cm^−1^ in the monomer crystal correspond to a translation along the molecular L inertia axis and to a rotation about the M axis, respectively. In the dimer they are found to combine contributing to the A_g_ modes calculated at 42.8 and 51.7 cm^−1^. While the former can still be described as a lattice phonon involving a rotation about the N inertia axis of the dimer, the latter becomes predominantly intramolecular in character and represents a first instance of the transformation of the nature (inter‐ to intra‐molecular) of the vibration. In the experimental Raman spectrum this accounts for the shift towards higher frequency, the intensity decrease and the broadening of the band observed in the monomer at 37.0 cm^−1^.

Similarly, the dimer crystal intramolecular A_g_ mode calculated at 56.5 cm^−1^, originates from both the monomer lattice translation at 59.5 cm^−1^ and the intramolecular vibration at 65.9 cm^−1^, whose contribution however spreads onto other modes of the product. Note that over this energy interval the experimental dimer Raman spectrum is given by the overlap of a number of broad bands, which are difficult to disentangle. A further instance of modes that map almost univocally from monomer to dimer is represented by the A_g_ pair that lies at 79.2 cm^−1^ (dimer) and 87.9 cm^−1^ (monomer), and its corresponding B_g_ pair (at 77.4 and 89.2 cm^−1^, respectively). The intermolecular contributions to these motions involve rotations of the monomeric units about their long inertia axis, pivoting, in the case of the dimer, on the four‐member ring and contributing to its broad main band. Indeed, in the wavenumber interval between 70 and 120 cm^−1^ of the monomer spectrum a very effective coupling of external and external degrees of freedom takes place, with external contributions that can be both rotations and translation and become intramolecular motions in the product lattice.

A better understanding of the dynamics evolution in this interval is given by the computation of the vibrational frequencies of the dimer molecule placed in the lattice of the monomer, that is of a system defined as [**1**_2_H_2_]Br_2_@[**1**H]Br_cell. Such a simulation has a twofold meaning: it makes the comparison between reactant and product volume independent by imposing the constancy of lattice parameters during the photoconversion process; it mimics the physical condition of a product which forms inside the lattice of the reactant, whose packing organization is thus preserved.

**Figure 5:**
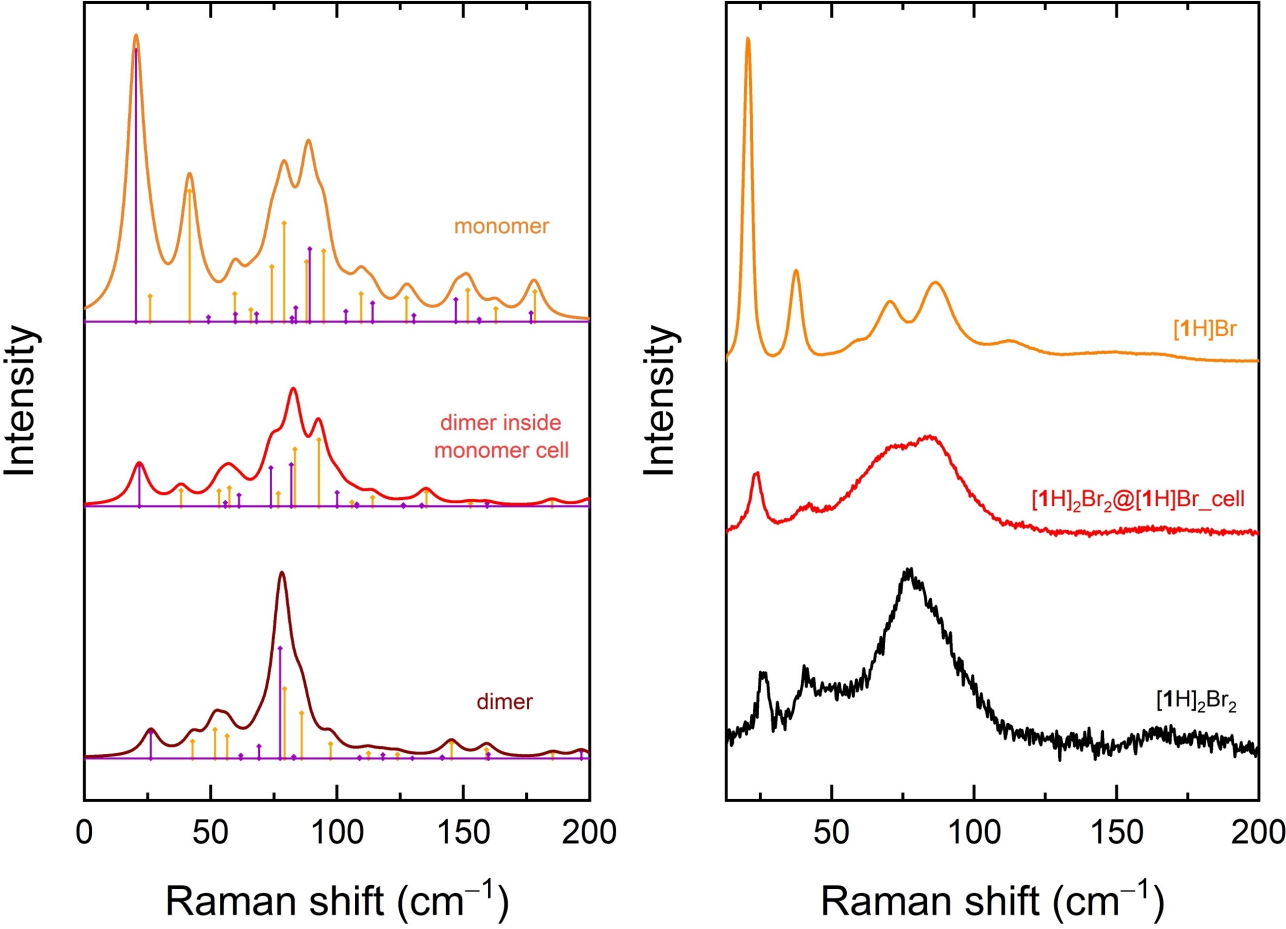
The low wavenumber simulated Raman spectrum of the dimer molecule located in the lattice of the monomer ([**1**_2_H2]Br_2_@[**1**H]Br) is compared to the corresponding spectra of pure [**1**H]Br and [**1**_2_H_2_]Br_2_ lattices (left) and to the experiments (right).

The low wavenumber Raman spectrum simulated for such a condition is displayed in Figure 5, where it is compared both to the simulations for the pure [**1**H]Br and [**1**_2_H_2_]Br_2_ lattices and to the corresponding experiments. There is no doubt that the spectrum of the [**1**_2_H_2_]Br_2_@[**1**H]Br_cell crystal bridges the differences between the spectra of the pure compounds and appears to account for many of the features displayed by the solid solutions. Accordingly, with few, localized exceptions, an almost perfect biunivocal correspondence between the vibrational modes of [**1**_2_H_2_]Br_2_ and [**1**_2_H_2_]Br_2_@[**1**H]Br_cell is found, substantiated by the dot product reported in Table S2 and graphically illustrated in Figure S3. This outcome is the most revealing of our approach, as it shows that the product not only can accommodate to the reactant lattice but also displays in it a vibrational dynamic, and therefore a phonon spectrum, which lies in between those of the pure systems. In fact, this reciprocal compatibility, which is not merely structural, accounts for the seamless evolution of the lattice phonon spectrum during the reaction and its simultaneity with the reaction itself.

It is worth noting that the calculations for the reverse operation, in which [**1**H]Br is projected onto the [**1**_2_H_2_]Br_2_ cell ([**1**H]Br@[**1**_2_H_2_]Br_2__cell) fails to attain a minimum energy condition and to produce a stable structure, most probably because of the smaller volume of the dimer cell. This suggests that whereas the reactant lattice can host the product molecule, the opposite might not hold.

## Conclusions

The intermediate states of a SCSC process such as that exhibited by the [2+2] photodimerization reaction of 4‐aminocinnamic acid salts are crystals of solid solutions in which the two components (reactant and product) are distributed in the lattice with an occupancy depending on the conversion degree. In this work, by applying low wavenumber Raman spectroscopy to the photoreaction of the model [**1**H]Br system, we demonstrate that such states display a clear spectral signature in the lattice phonon region, where the progress of the reaction is accompanied by a smooth evolution of the signal from reactant to product. In addition, we show that chemical and lattice transformations constitute in fact a single event.

Thus, the time evolution of the lattice phonon spectrum can effectively be employed to discriminate between homogenous and not homogenous^[16]^ reaction mechanisms. In the latter case, the seamless transition between spectra must be ruled out, while the formation of the product phase inside the parent reactant lattice usually occurs with an time delay with respect to the onset of the chemical reaction.^[16]^

Without attempting the extremely demanding task of computationally describing the vibrational states of the disordered systems represented by the solid solutions, we have employed DFT calculations to correlate [**1**H]Br monomer and dimer [**1**_2_H_2_]Br_2_ crystal normal modes. This has allowed us to better understand how the monotonical transformation of the spectrum of the reactant into that of the product is accomplished, by the mapping of each mode of the former onto one or more modes of the latter. Conceptually, the most intriguing result has been obtained in the calculation of the vibrational modes of the [**1**_2_H_2_]Br_2_ dimer molecule placed in the crystal structure of the monomer, as already highlighted in the previous Section, as the calculated low wavenumber Raman spectrum is compatible with those of the solid solutions, but the stability of this phase hints to the possible formation of a solid solution that would fully retain the packing arrangement of the starting material^[8]^ without any loss of the crystal integrity.

These findings suggest that the understanding of SCSC reactions can greatly be enriched from the study of their spectroscopic properties, with the support of targeted DFT calculations aiming to improve the description of solid solutions of large molecular systems.

## Experimental Section

The 4‐amino cinnamic acid (**1**) was purchased from Sigma‐Aldrich and was recrystallized from ethanol prior to use. Reagent grade solvents and bi‐distilled water were used. The salt [**1**H]Br (for its structure see Figure 1) was synthesized following the previously reported procedures:^[22]^ 200 mg (1.23 mmol) of 4‐aminocinnamic acid (**1**) were suspended in 5 mL of water and neutralized with HBr acid. The material undissolved was filtered off and the remaining solution was left to evaporate slowly in the dark.

Single crystals of [1H]Br (a few mm in size) were irradiated with a UV‐LED (LUXEON UV, total radiant flux ΦE=1100 W) at λ=365±5 nm placed at a distance of 1 cm from the sample to obtain the [12H2]Br2 dimer through a SCSC transformation.

Raman spectra of all the crystalline samples in the lattice phonon region (10–150 cm^−1^) were collected with a Horiba Jobin Yvon T64000 triple monochromator spectrometer interfaced with the optical stage of an Olympus BX40 microscope. The excitation wavelength was a Kr^+^ laser tuned at 647.1 nm set at very low power to avoid sample damage.

DFT simulations on the [**1**H]Br and its dimer crystals were performed using the code VASP (Vienna Ab initio Simulation Package).[^25^, ^26^, ^27^, ^28^] The Perdew‐Burke‐Ernzerhof (PBE) exchange correlation functional^[29]^ was employed together with projected‐augmented wave (PAW) pseudopotentials[^30^, ^31^] PAW_PBE H (15Jun2001), PAW_PBE C, PAW_PBE N, PAW_PBE O (08Apr2002) and PAW_PBE Br (06Sep2000). The effects of the dispersive interactions were included with the D3‐BJ pair‐wise correction by Grimme et al.^[32]^

A plane wave cutoff of 800eV proved to be adequate in combination with a 3×2×1 Monkhorst‐Pack k‐point sampling to achieve energy convergence for both structures. By raising the cutoff energy from 800 to 1200 eV, energy variations around 0.75 meV/atom were obtained, while the denser k‐point sampling of 10×7×3 gave energy changes below 0.006 meV/atom. Lower k‐point samplings actually yielded the same accuracy. The calculations were first performed at the experimentally determined unit cell parameters of each structure, relaxing the atomic coordinates until the residual forces fell below 1 meV/Å, using the GADGET package.^[33]^ Subsequently, the monomer molecular structure was projected into the experimental dimer unit cell and viceversa, thus relaxing again the atomic coordinates for both cases.

For all four minimized structures the vibrational modes, restricted to the Γ point, were computed at the corresponding fixed unit cell parameters with the force constants obtained by the PHONOPY software^[34]^ in combination with VASP. Polarizability tensors for each crystal mode were obtained by using the Python program vasp_raman.py,^[35]^ which uses the VASP code as backend. Raman intensities were finally adjusted by considering excitation wavelength and temperature dependence.

## Conflict of interest

The authors declare no conflict of interest.

1

## Supporting information

As a service to our authors and readers, this journal provides supporting information supplied by the authors. Such materials are peer reviewed and may be re‐organized for online delivery, but are not copy‐edited or typeset. Technical support issues arising from supporting information (other than missing files) should be addressed to the authors.

Supporting Information

## Data Availability

The data that support the findings of this study are available on request from the corresponding author. The data are not publicly available due to privacy or ethical restrictions.
