# Supplementary material for: Visualizing a Single‐Crystal‐to‐Single‐Crystal [2+2] Photodimerization through its Lattice Dynamics: An Experimental and Theoretical Investigation
Source: Chemphyschem. 2022 Apr 29;23(12):e202200168. doi: 10.1002/cphc.202200168 (PMC9325048; doi:10.1002/cphc.202200168)
Supplement: Supplementary file 1 — Supporting Information [file CPHC-23-0-s001.pdf]

ChemPhysChem

Supporting Information

Visualizing a Single-Crystal-to-Single-Crystal [2+2] Photodimerization through its Lattice Dynamics: An Experimental and Theoretical Investigation

Andrea Giunchi+, Lorenzo Pandolfi+, Tommaso Salzillo,* Aldo Brillante,
Raffaele G. Della Valle, Simone d'Agostino, and Elisabetta Venuti*

Supporting Information

Table of Contents

1. Lattice phonon Raman spectra of crystals obtained by irradiation of [**1**H]Br powder (λ = 365 nm) and recrystallization. 2

2. Calculated wavenumbers of the low energy Raman active modes of monomer [**1**H]Br, dimer [$\mathbf{1}_2H_2$]Br_2 and dimer inside the monomer cell [$\mathbf{1}_2H_2$]Br_2@[**1**H]Br_cell.. 3

3. SCSC transformation of a single crystal of [**1**H]Cl under irradiation (λ = 365 nm) investigated by Raman spectroscopy in the lattice phonon interval. 4

4. Graphical representation of the dot products between the vibrational eigenvectors of (a) [**1**H]Br monomer and [$\mathbf{1}_2H_2$]Br_2 dimer structures and (b) [$\mathbf{1}_2H_2$]Br_2 and [$\mathbf{1}_2H_2$]Br_2@[**1**H]Br_cell structures 6

5. Projections of [$\mathbf{1}_2H_2$]Br_2@[**1**H]Br_cell eigenvectors onto [$\mathbf{1}_2H_2$]Br_2 product dimer and [**1**H]Br reactant monomer........ 7

Author Contributions........ 7

1. Lattice phonon Raman spectra of crystals obtained by irradiation of [1H]Br powder (λ = 365 nm) and recrystallization.

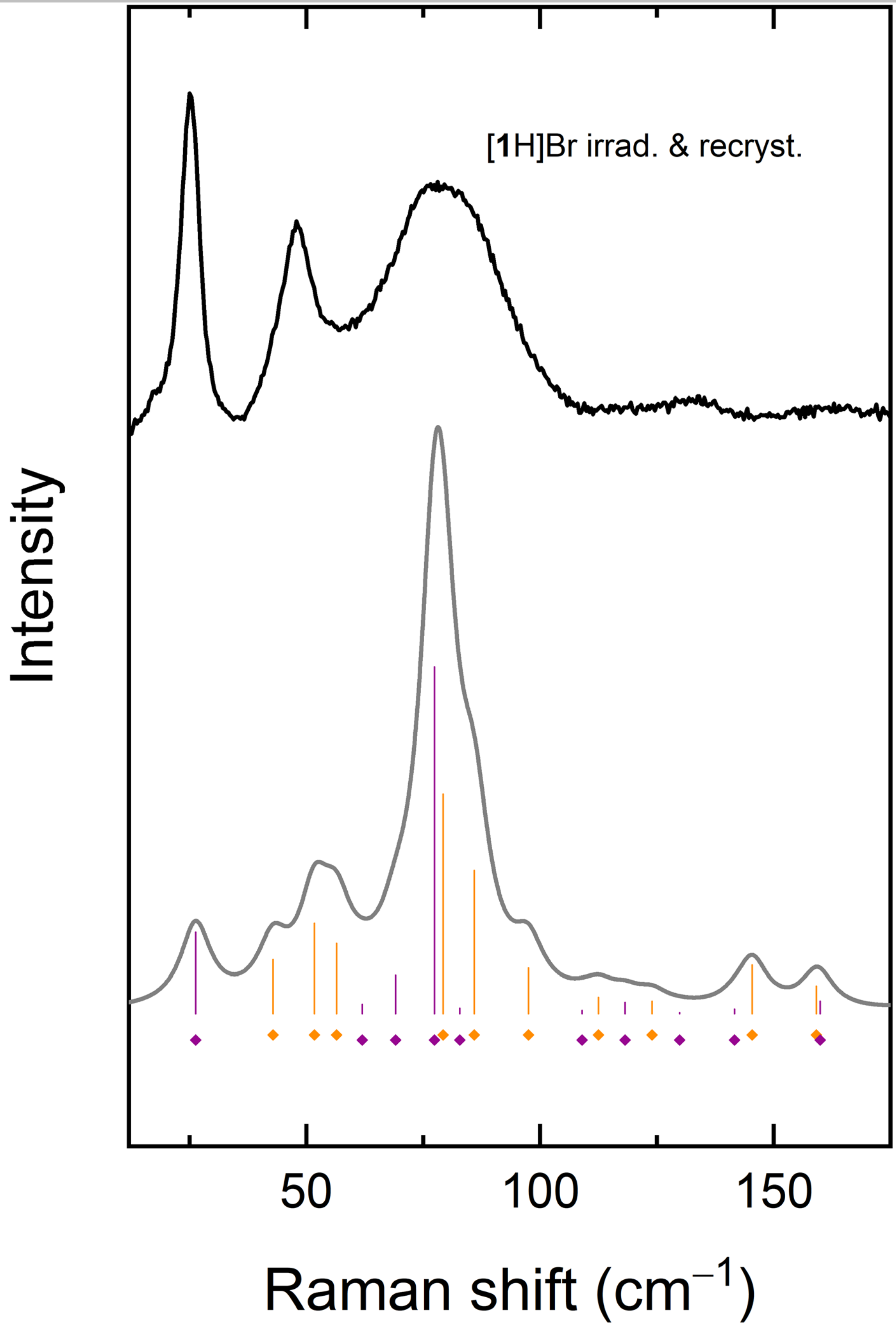

Figure S1. Lattice phonon Raman spectra of crystals obtained by irradiation of [**1**H]Br powder (λ = 365 nm) and recrystallization by solution evaporation (**black** line) compared to the DFT simulated spectrum of [**1**H]$_2$Br$_2$ (grey line). The vertical bars indicate the calculated positions of the peak values, with different colour codes to label the symmetry (yellow for A_g – violet for B_g) of the mode.

2. Calculated wavenumbers of the low energy Raman active modes of monomer [1H]Br, dimer [1$_2$H$_2$]Br$_2$ and dimer inside the monomer cell [1$_2$H$_2$]Br$_2$@[1H]Br_cell.

Table S1. Calculated wavenumbers of the low energy Raman active modes of monomer [**1**H]Br, dimer [$\mathbf{1}_2H_2$]Br_2 and dimer inside the monomer cell [$\mathbf{1}_2H_2$]Br_2@[**1**H]Br_cell. The calculated squared rotational components around the three inertia axes L, M, N (%R) for all systems, together with the squared translational components along the three inertia axes for the monomer (%T) account for the rigid body component of each vibration.

[$\mathbf{1}_2H_2$]Br_2			[$\mathbf{1}_2H_2$]Br_2@[1H]Br_cell			[**1**H]Br				
Freq (cm^{-1})	Sym	%R	Freq (cm^{-1})	Sym	%R	Freq (cm^{-1})	Sym	%T	%R	%T + %R
26.3	B_g	42	21.7	B_g	44	20.4	B_g	0	44	44
42.8	A_g	44	38.3	A_g	50	26.0	A_g	48	0	48
51.7	A_g	10	53.3	A_g	10	41.7	A_g	4	44	48
56.5	A_g	8	55.8	B_g	6	49.1	B_g	40	8	48
61.9	B_g	10	57.4	A_g	12	59.5	A_g	52	8	60
69.1	B_g	22	61.2	B_g	34	59.8	B_g	36	16	52
77.4	B_g	22	73.8	B_g	48	65.9	A_g	28	4	32
79.2	A_g	66	76.7	A_g	64	68.1	B_g	36	32	68
82.8	B_g	46	81.9	B_g	8	74.2	A_g	48	24	72
85.9	A_g	34	83.3	A_g	58	79.0	A_g	8	40	48
97.5	A_g	38	92.9	A_g	6	82.1	B_g	52	16	68
109.0	B_g	8	100.0	B_g	10	83.6	B_g	4	40	44
112.5	A_g	4	105.9	A_g	8	87.9	A_g	12	40	52
118.2	B_g	46	107.9	B_g	58	89.2	B_g	8	48	56
124.0	A_g	54	114.1	A_g	52	94.8	A_g	24	32	56
129.9	B_g	28	126.3	B_g	14	103.5	B_g	40	4	44
141.6	B_g	30	133.6	B_g	30	109.6	A_g	56	16	72
145.4	A_g	4	135.4	A_g	8	114.1	B_g	32	8	40
159.1	A_g	10	152.8	A_g	8	127.5	A_g	4	44	48
160.0	B_g	18	159.4	B_g	16	130.5	B_g	8	40	48
185.4	A_g	16	185.1	A_g	18	147.1	B_g	24	20	44
196.7	B_g	10	200.0	B_g	12	151.8	A_g	8	8	16

3. SCSC transformation of a single crystal of [1H]Cl under irradiation (λ = 365 nm) investigated by Raman spectroscopy in the lattice phonon interval.

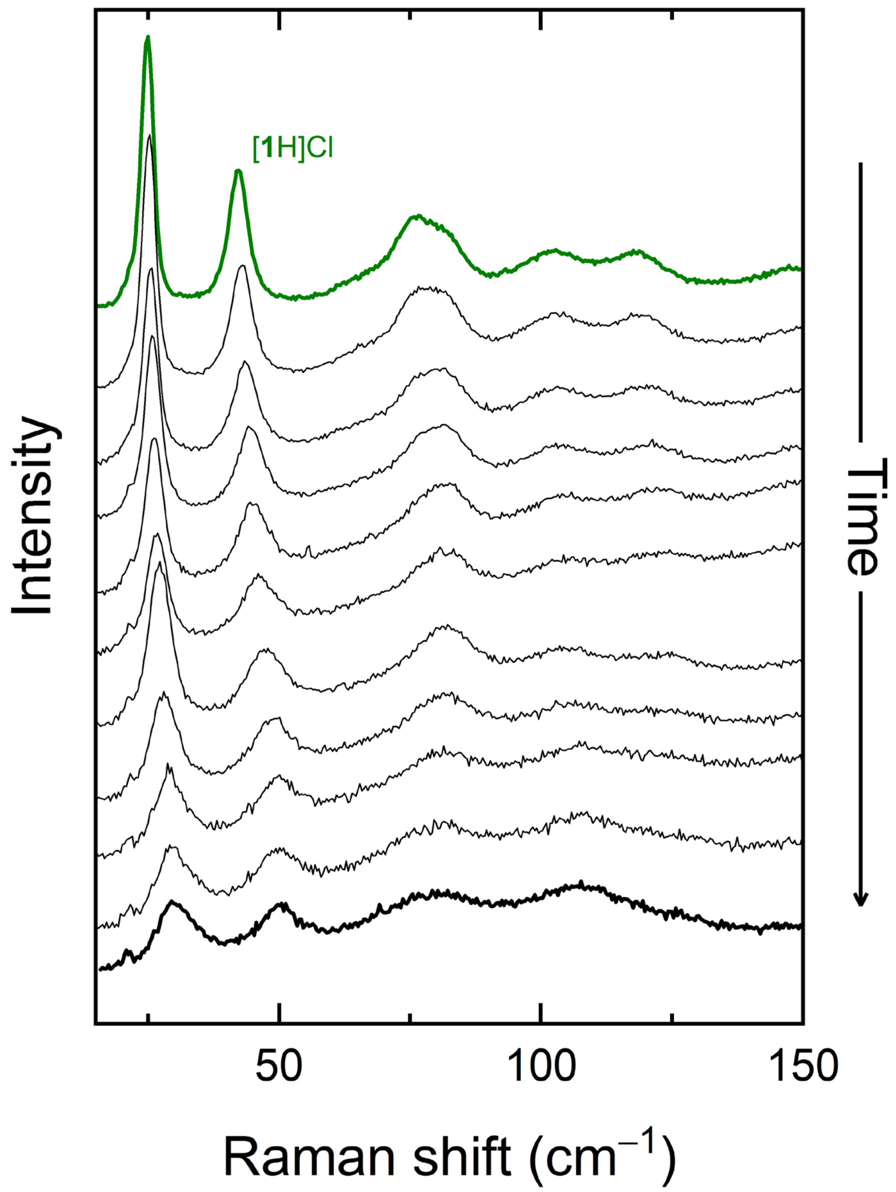

Figure S2. SCSC transformation of a single crystal of [**1**H]Cl under irradiation (λ = 365 nm) investigated by Raman spectroscopy in the lattice phonon interval. Spectra were recorded at time intervals of 15’. The green spectrum at the top identifies the pure monomer [**1**H]Cl; the **black** trace the pure dimer [$\mathbf{1}_2H_2$]Cl_2.

4. Graphical representation of the dot products between the vibrational eigenvectors of (a) [1H]Br monomer and [1_2H_2]Br_2 dimer structures and (b) [1_2H_2]Br_2 and [1_2H_2]Br_2@[1H]Br_cell structures

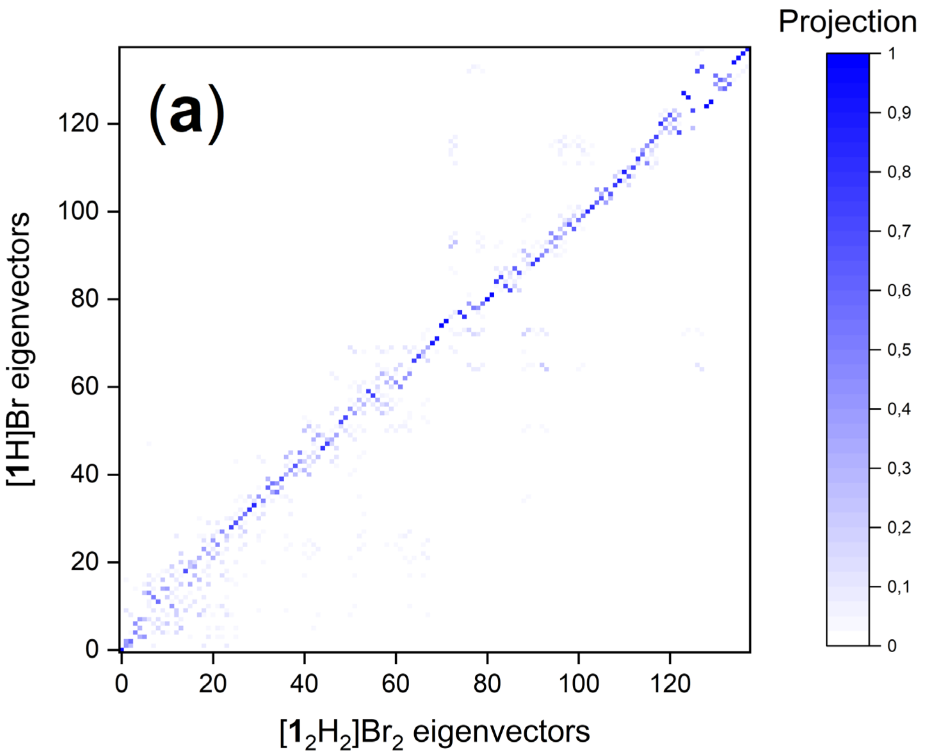

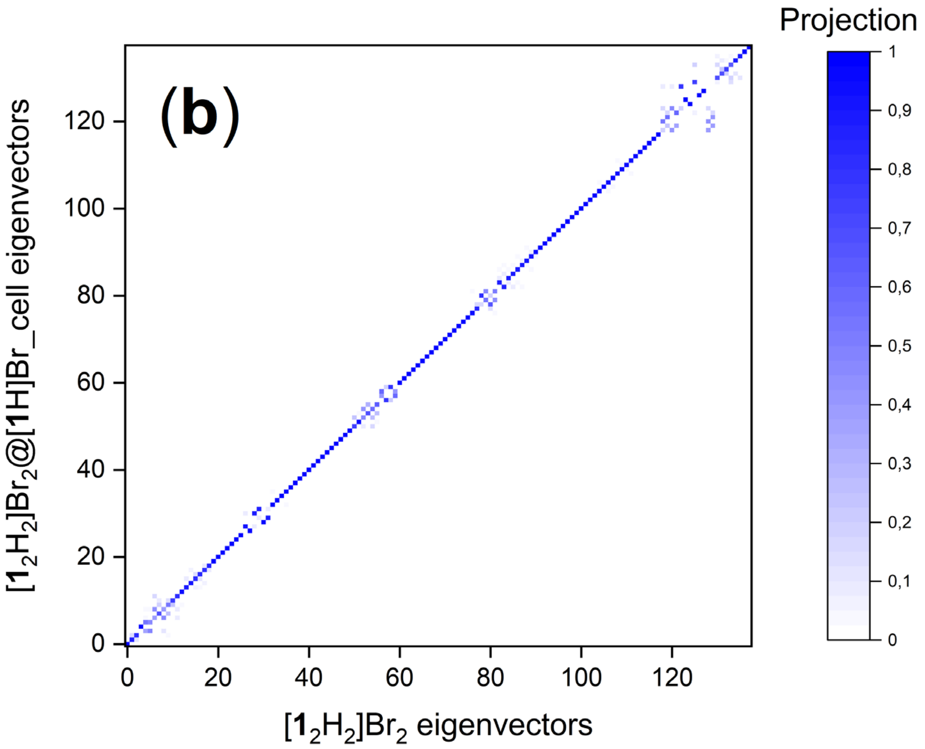

Figure S3. Graphical representation of the dot products between the vibrational eigenvectors of (a) [**1**H]Br monomer and [$\mathbf{1}_2H_2$]Br_2 dimer structures and (b) [$\mathbf{1}_2H_2$]Br_2 and [$\mathbf{1}_2H_2$]Br_2@[**1**H]Br_cell (i.e. the dimer molecule placed in the monomer lattice) structures, computed to determine how the vibrational dynamics of a structure maps onto the other. X and Y axes run over the wavenumbers in the lattice phonon region; the position of each point with respect to the diagonal measures the bi-univocal correspondence of the eigenvectors, while a color code is used to express quantitatively the overlap (between 0 and 1).

5. Projections of $[1_2H_2]Br_2$@[1H]Br_cell eigenvectors onto $[1_2H_2]Br_2$ product dimer and [1H]Br reactant monomer.

Table S2. Projections of $[1_2H_2]Br_2$@[**1**H]Br_cell eigenvectors onto those of $[\mathbf{1}_2H_2]Br_2$ product dimer (left) and [**1**H]Br reactant monomer (right). The dot products are reported in bracket for the modes with the highest contributions, with the sum of the contributions set to be ≥ 0.5. In bold the frequency of the mode which represents the best match with the one of $[1_2H_2]Br_2$@[1H]Br_cell.

**Freq** $[\mathbf{1}_2H_2]Br_2$ (cm^{-1}) and projection $[\mathbf{1}_2H_2]Br_2$ ← $[\mathbf{1}_2H_2]Br_2$@[1H]Br_cell		**Freq** $[\mathbf{1}_2H_2]Br_2$@[1H]Br_cell (cm^{-1})	Sym		**Freq** [1H]Br (cm^{-1})and projection $[\mathbf{1}_2H_2]Br_2$@[1H]Br_cell → [1H]Br
**26.3** (1.00)	←	**21.7**	B_g	→	**20.4** (.97)
**42.8** (.85)	←	**38.3**	A_g	→	**41.7** (.67)
**51.7** (.82)	←	**53.3**	A_g	→	**26.0** (.44), 41.7 (.22)
61.9 (.37), **69.1** (.48)	←	**55.8**	B_g	→	**49.1** (.64)
**56.5** (.97)	←	**57.4**	A_g	→	**59.5** (.51)
**61.9** (.53)	←	**61.2**	B_g	→	**68.1** (.59)
77.4 (.40), **82.8** (.47)	←	**73.8**	B_g	→	**83.6** (.53)
**79.2** (.69)	←	**76.7**	A_g	→	**87.9** (.37), 65.9 (.23)
82.8 (.39), **77.4** (.44)	←	**81.9**	B_g	→	**89.2** (.31), 68.1 (.20)
**85.9** (.53)	←	**83.3**	A_g	→	**94.8** (.73)
**97.5** (.76)	←	**92.9**	A_g	→	**87.9** (.32), 65.9 (.18)
**109.0** (.81)	←	**100.0**	B_g	→	**82.1** (.46), 114.1 (.27)
**112.5** (.93)	←	**105.9**	A_g	→	**74.2** (.17), 59.5 (.16), 109.6 (.15), 79.0 (.15)
**118.2** (.89)	←	**107.9**	B_g	→	**147.1** (.36), 156.3 (.15)
**124.0** (.92)	←	**114.1**	A_g	→	**127.5** (.65)
**129.9** (.80)	←	**126.3**	B_g	→	**103.5** (.45), 147.1 (.18)
**141.6** (.84)	←	**133.6**	B_g	→	**130.5** (.60)
**145.4** (.82)	←	**135.4**	A_g	→	**151.8** (.27), 109.6 (.19), 74.2 (.15)
**159.1** (.87)	←	**152.8**	A_g	→	**162.9** (.36), 74.2 (.14), 227.9 (.09)
**160.0** (.98)	←	**159.4**	B_g	→	**156.3** (.22), 103.5 (.19), 276.3 (.15)
**185.4** (.97)	←	**185.1**	A_g	→	**151.8** (.38), 178.3 (.34)
**196.7** (.97)	←	**200.0**	B_g	→	**176.9** (.47), 156.3 (.25)
**210.1** (.99)	←	**208.3**	B_g	→	**236.1** (.45), 82.1 (.13)
**219.9** (.97)	←	**218.6**	A_g	→	**227.9** (.19), 178.3 (.17), 26.0 (.11), 74.2 (.09)
**256.0** (.96)	←	**243.7**	A_g	→	**273.2** (.69)
**269.2** (.96)	←	**255.3**	B_g	→	**276.3** (.54)

Author Contributions

Andrea Giunchi (investigation, equal contribution), Lorenzo Pandolfi (investigation, equal contribution), Tommaso Salzillo (validation), Aldo Brillante (validation), Raffaele G. Della Valle (Formal analysis), Simone D'Agostino (validation) and Elisabetta Venuti (lead the project, validation, writing original draft)
